# Recent progress in the study of brown adipose tissue

**DOI:** 10.1186/2045-3701-1-35

**Published:** 2011-10-28

**Authors:** Xuan Yao, Shifang Shan, Ying Zhang, Hao Ying

**Affiliations:** 1 Key Laboratory of Nutrition and Metabolism, Institute for Nutritional Sciences, Shanghai Institutes for Biological Sciences, Chinese Academy of Sciences, Shanghai, China; 2 Graduate School of the Chinese Academy of Sciences, Chinese Academy of Sciences, Shanghai, China

**Keywords:** brown adipose, differentiation, thermogenesis, obesity control

## Abstract

Brown adipose tissue in mammals plays a critical role in maintaining energy balance by thermogenesis, which means dissipating energy in the form of heat. It is held that in mammals, long-term surplus food intake results in energy storage in the form of triglyceride and may eventually lead to obesity. Stimulating energy-dissipating function of brown adipose tissue in human body may counteract fat accumulation. In order to utilize brown adipose tissue as a therapeutic target, the mechanisms underlying brown adipocyte differentiation and function should be better elucidated. Here we review the molecular mechanisms involved in brown adipose tissue development and thermogenesis, and share our thoughts on current challenges and possible future therapeutic approaches.

## Introduction of brown adipose tissue and thermogenesis

Adipose depots in different parts of the body have unique micromorphology and molecular markers. According to their distinct physiological roles, adipose tissue in mammals is categorized into white adipose tissue (WAT) and brown adipose tissue (BAT) [1]. WAT functions to store energy in the form of triglyceride (TG)-containing intracellular droplets as well as to secrete a host of hormones that regulate overall energy balance. Unlike WAT, BAT regulates thermogenesis upon environmental stresses to maintain energy balance and protect the organism from hypothermia [2]. In addition, it has been shown that by activating BAT via short-term cold exposure, fatty acids are efficiently channeled into BAT due to a metabolic program that boosts TG-rich lipoproteins (TRL) uptake into BAT. In consequence, lipids clearance from plasma becomes more efficient, implicating that BAT might be a master regulator of TRL clearance and blood lipid abundance [3].

BAT thermogenesis is achieved by dissipating heat produced from fatty acid oxidation. As little as 50 g of BAT could account for up to 20% of basal metabolic energy expenditure of an adult human when maximally stimulated [4]. When animals are subjected to cold environment or ingest surplus energy, their sympathetic nervous system will be stimulated and catecholamine will be released. Catecholamine binds to the β3-Adrenoceptor on the plasma membrane of brown adipocyte and activates adenylyl cyclase which is able to accelerate the conversion of ATP to cyclic AMP (cAMP) [5]. cAMP is able to activate type 2 deiodinase (Dio2), an enzyme that converts thyroid hormone T4 to T3 in the brown adipocyte, resulting in enhanced local thyroid hormone signaling and increased energy. In addition, cAMP activates protein kinase A, which, in turn, phosphorylates and activates triacylglycerol lipase. Then the lipase accelerates the release of free fatty acid from triacylglycerol contained in the droplet of brown adipocyte, which acts both as the fuel of thermogenesis and as an activator of uncoupling protein 1 (UCP1), a key component of thermogenesis. UCP1 activation results in fast substrate oxidation with a low rate of ATP production. Thus, a large amount of energy is dissipated in the form of heat, which will be distributed throughout the body by circulation system [1,6].

## Overview of recent breakthroughs in the study of BAT

It was previously believed that brown adipocyte and white adipocyte share a common ancestor in the course of adipogenesis. However, different opinion was rendered in 2007 when PR domain containing 16 (PRDM16) was discovered to be a master regulator of brown adipocyte differentiation [6]. Investigation of its specific role in brown adipocyte commitment led to the discovery that skeletal muscle and some depots of BAT share a common myogenic factor 5 (Myf5)-expressing progenitor [7]. Besides classic interscapular brown adipocytes, brown-like adipocytes (brite adipocytes) were found in WAT depots. When rats were submitted to cold or treated with β-adrenoceptor agonist, UCP1 could be detected in fat pads which had been considered as WAT. UCP1 was expressed in cells morphologically identical to typical brown adipocytes found in interscapular BAT [8]. More recent data suggested that there were inducible brown adipocytes in WAT when mice were exposed to cold environment [9]. These inducible brown adipocytes were not derived from Myf5-expressing myoblasts, and had a distinct gene expression profile in comparison with those adipocytes in interscapular BAT.

**Figure 1:**
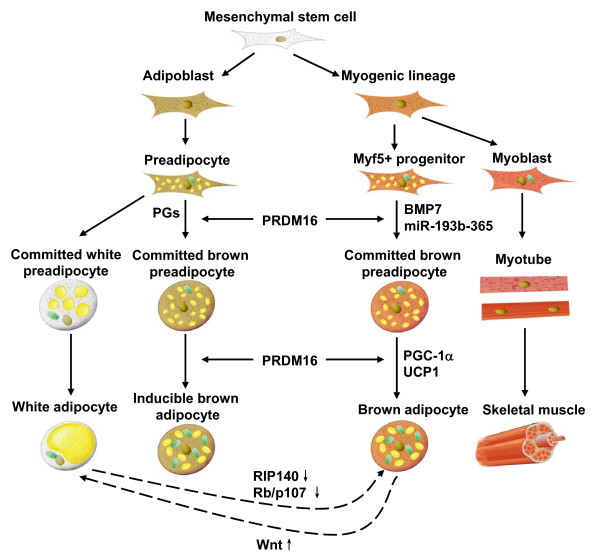
**Schematic representation of the effect of key regulators involved in brown adipose differentiation**.

The mesenchymal stem cell is the precursor of brown adipocytes. BAT formation occurs in the early phase of embryonic development [10]. Once it was suggested that only small mammals and new born infants possess BAT, while human adults are practically devoid of functional BAT [1]. However, several research papers published in *New England Journal of Medicine *in 2009 rebutted this assumption [11-14]. After analyzing thousands positron emission computed tomography/computed tomography (PET/CT) scans of adult subjects, researchers found that BAT was located in the neck and upper-chest regions. Moreover, BAT ratio was conversely correlated with body mass index (BMI) and this correlation was more significant among elderly [12]. Development of obesity is a result of prolonged positive energy balance. Most of surplus energy is stored in adipose tissue. Since BAT is essential in maintaining the balance of body fat and keeping individuals from obesity, now BAT has been considered a promising therapeutic target to combat obesity. We need to further delineate the mechanisms involved in brown adipose differentiation and thermogenesis, although they have been studied extensively (Figure 1).

## Key players involved in brown adipose thermogenesis and differentiation

### UCP1

As for human, UCP1 genetic polymorphisms were reported to be correlated with fat metabolism, obesity and diabetes [15-17]. These genetic studies underlie the role of UCP1 in maintaining energy balance. UCP1 is predominantly expressed in brown adipocytes and responsible for the respiratory uncoupling during BAT thermogenesis [1]. Located on the inner membrane of mitochondria, UCP1 activity is regulated at multiple levels. There exist peroxisome proliferator responsive element (PPRE) [18], thyroid hormone responsive element (TRE) [19], and retinoic acid responsive element (RARE) [20] in the upstream of UCP1 gene, which means these nuclear receptors and their ligands might directly regulate its transcription. In addition, peroxisome proliferator-activated receptor gamma coactivator 1-alpha (PGC-1α), which is critical to mitochondria genesis, is able to increase the transcriptional activity of peroxisome proliferator-activated receptor γ (PPARγ) and thyroid hormone receptor (TR) on the UCP-1 promoter [21]. UCP1 activity can also be directly regulated by purine nucleotides and free fatty acids. Purine nucleotides bind from the cytosolic side of UCP1 and inhibit its activity [22]. Free fatty acids released in response to stimuli can replace the purine nucleotides and act as a UCP1 activator [23,24]. After the residual fatty acids are oxidized away, the mitochondria will return to coupled state.

### Thyroid hormone and Dio2

Thyroid hormone is necessary for a full BAT thermogenic response in cold adaptation. In the hypothyroid state, the response of BAT to sympathetic stimulation is diminished [25]. Mice deficient in TRα are cold intolerant and have impaired BAT thermogenic response to norepinephrine [26]. In addition, activation of thyroid hormone signaling is highly regulated by deiodination. Deiodination of minimally active thyroxine (T4) to the biologically active triiodothyronine (T3) is catalyzed by type I and type II deiodinases (Dio1 and Dio2) [27]. Dio2 expression has been shown to control intracellular T3 concentration. In response to sympathetic stimulation, increased cAMP stimulates T3 production by increasing Dio2 expression and activity, which leads to increased energy expenditure in BAT. Mice lacking Dio2 exhibit permanent BAT thermogenic defect, compromising thermoregulation and the ability to dissipate excessive calories from diet [28-30]. Interestingly, bile acids released from the gallbladder has been shown to increase energy expenditure in BAT by regulating local thyroid hormone production via G protein-coupled receptor TGR5-cAMP-Dio2 signaling pathway [31,32].

### PGC-1α

Before the identification of PRDM16 in brown adipocytes, PGC-1α was considered as a major regulator in brown adipocyte function. PPARγ is a nuclear receptor critical for both WAT and BAT formation, which was evidenced by the substantial studies in knockout mice [33]. PGC-1α, discovered as a coactivator for PPARγ, is believed to determine the specific role of PPARγ to promote UCP1 transcription in brown adipocytes [21]. PGC-1α content is higher in BAT than in WAT. PGC-1α expression increases in the course of brown adipocyte differentiation [34]. Moreover, PGC-1α can induce the expression of nuclear respiratory factor (NRF) and physically interact with NRF to coactivate the transcription. NRF is able to regulate a host of mitochondrial genes encoded in the cell nucleus, including β-ATP synthase, cytochrome-c, cytochrome-c-oxidase subunit IV, and mitochondrial transcription factor A [35]. Cold exposure significantly stimulates PGC-1α expression, which is mediated by protein kinase A-cAMP response element-binding protein (PKA-CREB) pathway [21]. Ectopic expression of PGC-1α in WAT leads to mitochondria genesis and UCP1 upregulation. Lack of PGC-1α in mice does not affect the morphology of BAT but results in lower UCP1 level and intolerance to cold [36,37]. Correspondingly, loss of PGC-1α in cultured brown preadipocytes does not impede the maturation, but the matured adipocytes have impaired thermogenesis [34]. Thus, it can be concluded that PGC-1α is an important part of thermogenesis due to its indispensable role in mitochondria genesis while it has limited effect on brown adipocyte differentiation.

### RIP140

Receptor-interacting protein 140 (RIP140), a nuclear receptor corepressor, can suppress nuclear receptor estrogen receptor (ER) and PPAR in the presence of their ligands [38]. It is more abundantly expressed in WAT than in BAT [39,40]. It was reported that RIP140 deficiency in cultured adipocytes led to increased energy expenditure and this phenomenon disappeared if RIP140 was re-expressed [41]. *In vivo *studies show that RIP140^-/- ^mice consume a similar amount of food as control mice, while their physical activity remains unaltered. The mice appear to be emaciated with a total fat tissue content dropped to 30% of that in control mice. However, in these mice, the fat cell number remains the same compared to the control group, but the volume decreases significantly. Moreover, the RIP140^-/- ^mice are protected from high-fat diet-induced obesity. It is of particular interest that UCP1 expression in the WAT of these mice is 100 times higher than that in the WAT of wild type mice [39]. This finding suggests that RIP140 might be able to suppress brown adipocyte characteristics.

### PRDM16

PRDM16, a 140-kDa protein, contains an N-terminal PR domain, ten zinc-fingers and other diverse sites that can mediate multiple protein-protein interaction. PRDM16 plays a critical role in determining brown adipocyte lineage commitment and differentiation. By comparing the transcriptome of BAT and WAT from mice, PRDM16 was for the first time recognized as a BAT-specific gene [6]. When PRDM16 is expressed in white fat cell progenitors, it can activate a robust brown fat phenotype including induction of PGC-1α, UCP1, and Dio2 expression and a remarkable increase in uncoupled respiration. Transgenic expression of PRDM16 in white fat depots stimulates the formation of brown fat cells. In contrast, knockdown of PRDM16 through shRNA expression in brown fat cells causes a near total loss of the brown characteristics. It is suggested that PRDM16 activates brown fat cell identity at least in part by simultaneously activating PGC-1α and PGC-1β through direct binding [6], and represses white adipocyte gene expression through forming complex with C-terminal-binding protein-1 (CtBP-1) and CtBP-2 [42].

A major breakthrough was achieved by a lineage tracing study *in vivo *which has revealed that brown adipocyte shares a mutual precursor, Myf5-expressing cell, with skeletal muscle cell, but not white adipocyte as previously considered. Loss of PRDM16 from brown adipocyte precursor promotes muscle differentiation, while ectopic expression of PRDM16 in myoblasts induces their differentiation into brown adipocytes [7]. Subsequent investigation indicates that PRDM16 is able to form complex with CCAAT-enhancer-binding proteinβ (C/EBPβ) and this complex controls the cell fate switch from myoblastic precursors to brown fat cells [43]. Forced expression of PRDM16 and C/EBP-β is sufficient to induce a fully functional brown fat program in naive fibroblastic cells. All the evidence above demonstrates that PRDM16 plays a critical role in determining the differentiation fate of brown adipocyte. However, the specific upstream regulator of PRDM16 remains to be elucidated.

Recently, PRDM16 was also proved to be important in determining the thermogenic program of subcutaneous WAT [44]. Transgenic expression of PRDM16 in fat tissue robustly induces the development of brown-like adipocytes in subcutaneous, but not epididymal, adipose depots. PRDM16 transgenic mice display increased energy expenditure. These findings indicate that PRDM16 is a key regulator for brown fat-like gene program and thermogenesis in subcutaneous adipose tissues.

### Wnt

The Wnt proteins are a group of autocrine or paracrine secreted glycoproteins [45]. Over ten members have been uncovered in the Wnt family, all of which participate in the regulation of embryonic development and maintenance of adult tissue homeostasis. BAT expresses Wnt10a and Wnt10b. Wnt proteins are found to be downregulated in the course of brown adipocyte formation, suggesting that these Wnt proteins inhibit the maturation of brown adipocytes [46]. To investigate the mechanism underlying the effect of Wnt10b in adipocyte formation *in vivo*, Longo et al. constructed transgenic mice which express Wnt10b specifically in adipose tissue, named as FABP4-Wnt10b mice [47]. Their investigation discovered that the BAT development in FABP4-Wnt10b mice is impaired and the appearance of interscapular BAT resembles that of WAT. Moreover, the BAT function is deficient, which is manifested by the fact that the transgenic mice are not able to maintain core body temperature when exposed to cold environment [46,47]. *In vitro *study reported by Kang et al. shows that PPARγ and C/EBPα are involved in the inhibition of brown adipocyte differentiation caused by Wnt10b, while the suppression of UCP1 expression is related to PGC-1α [46]. To specifically express Wnt10b in BAT, they constructed transgenic mice in which Wnt10b transcription is under the control of UCP1 promoter (UCP1-Wnt10b mice). By investigating the histological morphology of the interscapular BAT, they found that each adipocyte contains single lipid droplet, which should have existed only in white adipocytes. In comparison with BAT of control mice, protein levels of UCP1 and PGC-1α in UCP1-Wnt10b mice decrease greatly with the suppression of mitochondria genesis and metabolism, yet the expression of PPARγ and C/EBPα remains unaltered. As a result, they concluded that in mature brown adipocytes, enhanced Wnt signaling pathway endows brown adipocytes with significant characters of white adipocytes. Moreover, reciprocal expression of Wnt10b with UCP1 and PGC-1α in interscapular BAT from cold-challenged or genetically obese mice provides further evidence for regulation of brown adipocyte metabolism by Wnt signaling [46]. Based on these results, we believe that the Wnt signaling pathway inhibits maturation of brown preadipocyte and suppresses the characters of mature brown adipocytes.

### Pocket protein

Pocket proteins play key roles in cell cycle progression [48,49]. They can bind to E2F family and inhibit the transcription of target genes [50]. Pocket proteins include retinoblastoma protein (pRb), p107 and p130. pRb has been shown to influence adipocyte differentiation. When embryonic stem cells of wild type and Rb^-/- ^mice are induced to differentiate into adipocytes, UCP1 will be exclusively expressed in Rb^-/- ^adipocytes. In addition, Rb^-/- ^adipocytes have higher expression level of PGC-1α. Mouse embryonic fibroblast (MEF) is widely used to study the process of adipocyte differentiation *in vitro*. When MEFs are induced to differentiate into adipocytes, UCP1 is elevated only in Rb^-/- ^MEF but not in wild type MEF, and the PGC-1α level is much higher in the former one. In the mature adipocytes, the expression level of UCP1 and PGC-1α is comparable to that of wild type BAT. Observation of the electron microscopic samples revealed that pRb-deficient adipocytes have more mitochondria than the control group [51]. In addition, Rb^-/- ^adipocytes have higher levels of forkhead box protein C 2 (Foxc2) and regulatory type I alpha (RIα) during differentiation as compared to the wild type. Further studies revealed that Foxc2 is indispensable to RIα activation, whereas the activated RIα is able to enhance cAMP sensitivity because RIα has very high affinity to cAMP. Enhanced cAMP sensitivity is critical in the early phase of adipocyte differentiation for the reason that it can activate CREB and induce the expression of PGC-1α, thus increases UCP1 expression level and mitochondria genesis. Alteration of p107, another member of the pocket proteins, can also affect the differentiation of adipocytes. As reported by Scime et al. in 2005, WAT of p107^-/- ^mice are totally replaced by BAT [52]. Each adipocyte in WAT contains multilocular lipid droplets with PGC-1α and UCP1 expression levels similar to that in BAT. Additionally, pRb level decreases in WAT of p107^-/- ^mice. To be noted, pRb is able to suppress the transcription of PGC-1α by binding to its promoter. Along this line of evidence, it can be concluded that PGC-1α is the target of p107 and pRb in preadipocytes.

### Prostaglandins

Prostaglandins are important biological mediators derived from fatty acids. They are composed of twenty carbon molecules, five of which make up a five-member ring. Cyclooxygenase (COX) is the enzyme catalyzing the committed step in prostaglandin synthesis. At present, two COX isoenzymes, COX-1 and COX-2, have been well studied. They act in a similar fashion in the catalytic reaction [53]. COX-1 is considered as a constitutive enzyme, being found in most mammalian cells. COX-2, on the other hand, is only detectable in specific tissues when inflammation happens. Being an inducible enzyme, COX-2 becomes abundant in activated macrophages and other cells at sites of inflammation [54,55]. Besides being critical mediators in inflammation, prostaglandins and COX are also found to play a role in maintaining energy balance. Mice heterozygous for COX-2 tend to be obese. Of great interest is the result reported by Vegiogoulos A et al. in 2010 that COX-2 is one of the targets of adrenaline stimulation in subcutaneous WAT and critical to the induction of brown adipocytes in WAT [9]. Treatment of prostaglandin, the product of COX-2 catalyzed reaction, is able to promote committed mesenchymal stem cells to differentiate into adipocytes with brown fat character. Moreover, overexpression of COX-2 in WAT of mice effectively induces *de novo *recruitment of brown adipocytes, elevates the energy expenditure of mice and protects the mice from high fat diet induced obesity. Before long, this conclusion was confirmed by Madsen A et al [56]. These data strongly indicate that COX-2 is critical for the UCP1 expression in brown adipocytes recruited in WAT.

### BAT and Obesity Treatment

**Figure 2:**
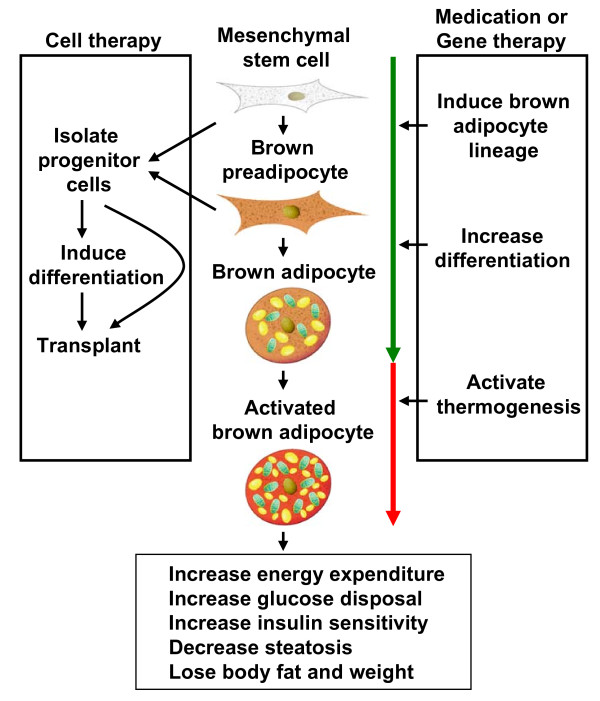
**Schematic representation of two BAT-based therapeutic strategies in obesity control**.

Obesity is defined not only as an excess of body weight, but also an increased adipose tissue accretion to the extent that health may be adversely affected. The anti-obesity medication phentermine and orlistat approved by Food and Drug Administration (FDA) are designed to suppress appetite and reduce fat absorption, respectively [57]. Unfortunately, phentermine has adverse effects including increased heart rate and elevated blood pressure, while orlistat may cause steatorrhea, fecal incontinence and frequent or urgent bowel movements. Due to the presence of BAT in adult humans, it is conceivable that weight loss can be achieved by increasing energy expenditure through activating BAT. Until now, two therapeutic strategies have been suggested in obesity control as shown in Figure 2[58,59]. One is to stimulate the original BAT development and function by small molecules. The other is to transplant functional brown adipocytes induced from proper stem cells into obese patients. The second strategy can be also considered as BAT transplantation.

Dinitrophenol (DNP) was developed to uncouple the biological process of oxidative phosphorylation in mitochondria and make mitochondria produce heat instead of ATP [60]. It increases energy expenditure in a sustainable manner and no drug tolerance would happen. Although DNP has been withdraw from the market due to the side effects, including a sensation of warmth, frequently with sweating and hyperthermia, chemical modification will hopefully help us develop a better drug [61]. A theory concerning human body weight suggests that there is a set point of body weight. Owning to the concurring effort of nerve and endocrine system, body weight will fluctuate in a narrow window. In other words, normal body weight can be regained even if short term energy intake (or expenditure) that leads to increased (or decreased) weight. However, under long term one-way stimulation, this "set point" can be resettled. This provides a possible therapy for the obese by treating them with BAT activating drugs so as to lower their set point.

BAT transplantation becomes increasingly appealing due to the gradually perfection of stem cell technology. Adipose tissue transplantation has primarily been used for human reconstructive surgery. Now, transplantation of adipose tissue is being explored as a possible tool to promote the beneficial metabolic effects of subcutaneous WAT and BAT, as well as adipose-derived stem cells [62]. Brown adipocytes at varied differentiation stages or the complete BAT can be transplanted. Cells that can be transplanted include stromal vascular fraction (SVF), preadipocytes and brown adipocytes. Furthermore, confluent preadipocytes cultured in dishes, dedifferentiated primary adipocytes and SVF show better viability than the fully differentiated adipocytes and mature adipocytes. It is worth mentioning that the success rate of transplantation is partially determined by the degree of vascularization [63].

### Problems and Perspectives

Rediscovery of BAT in human adults brought about the revival of BAT research as an anti-obesity therapy. Despite the growing number of research results reported, there are many unanswered questions on BAT development and thermogenesis, which may be better understood with appropriate animal models. Animals expressing Cre recombinase under the control of UCP1 promoter have not yet been widely applied in the research of BAT. In addition, negligence of the temperature under which mice are raised may also mislead the data analysis. 30°C is the thermoneutral temperature for mice, at which mice maintain their body temperature without adaptive nonshivering thermogenesis. However, mice are usually bred at 22°C, a temperature that UCP1 can be activated during BAT thermogenesis. As a result, behaviors exhibited by knockout mice under this circumstance may not solely reflect the gene function. In addition to environmental temperature, individual variance of hair density and physical activity, which influence body temperature and energy expenditure, will also incur error when explaining the phenotypes observed from mice.

Currently, most efforts are spent to delineate signaling pathways and transcriptional regulation. Emerging areas like RNA editing, alternative splicing, non-coding RNA and epigenetic modification are largely unexplored. During the preparation of this review, miR-193b-365, a brown-fat-enriched miRNA cluster, was identified as a key regulator of brown fat development [63]. Since the BAT development and function are highly orchestrated and complex processes, regulatory mechanisms involved deserve more attention.

## Conclusions

With the help of modern technology, multiple studies conclusively show that functional BAT exists in adult humans, and is inversely correlated with BMI, adipose tissue mass, glucose and insulin levels. Recently, studies were focused on illustrating which factors determine the unique feature of BAT. As summarized above, a great number of molecules are involved in the regulation of brown adipocyte differentiation and thermogenesis, be it in direct or indirect ways. Animal studies contribute a great deal for us to understand BAT development and function. However, a full understanding of BAT biology in humans will only be completed with clinical evidence. Taking advantage of the advancement of biomedical technology, we are expecting the next leap on BAT research.

## List of abbreviations used

WAT: white adipose tissue; BAT: brown adipose tissue; TG: triglyceride; TGL: TG-rich lipoproteins; UCP1: uncoupling protein 1; cAMP: cyclic AMP; PRDM16: PR domain containing 16; Myf5: myogenic factor 5; PET/CT: positron emission computed tomography/computed tomography; BMI: body mass index; PGC-1α: Peroxisome proliferator-activated receptor gamma coactivator 1-alpha; PPAR: peroxisome proliferator-activated receptor; NRF: nuclear respiratory factor; CREB: cAMP response element-binding protein; RIP140: receptor-interacting protein 140; Dio2: type II deiodinase; ctBP: C-terminal-binding protein; C/EBP: CCAAT-enhancer-binding protein; pRb: retinoblastoma protein; MEF: mouse embryonic fibroblast; COX: cyclooxygenase; DNP: 2, 4-dinitrophenol; SVF: stromal vascular fraction.

## Competing interests

The authors declare that they have no competing interests.

## Authors' contributions

XY wrote the manuscript, SS, YZ, and HY revised the manuscript. All authors read and approved the final manuscript.
